# Some Uses of Paraffin for Chemical Purposes

**Published:** 1862-07

**Authors:** 


Some Uses of Paraffin for Chemical Purposes.—The price of paraffin keeps step with that of the other products of the distillation of coal, tar, and petroleum, and being now about as low as it will probably ever come to, there will not be wanting many important uses for it, especially in the chemical and pharmaceutical line. That it is acted on neither by chlorine, nor by caustic alkalies, nor by acids, has given to it its name, though it may be as well to notice that the non-action of oil of vitriol refers only to the pure article, and that inasmuch as all, or very nearly all, the paraffin sold here contains a certain proportion of stearic acid, which is added for some good and some bad reasons, it will be darkened more or less by the strong acid. Some of its applications, so far as we can call them to mind at present, we will mention. A great inconvenience in chemical analysis and other manipulations is the use of oil or tallow, and metallic baths for obtaining constant temperatures higher than the boiling point of water and solution of salts—the fats, on account of the production of acrolein and similar unpleasant exhalations, and the metals, as well as the salts, by the formation of crusts of oxides, etc. on the surface. Paraffin, even when not altogether pure, will give any constant temperature up to 550° F., which can be kept up by a spirit-lamp or a gas-lamp, with a contrivance like Kemp's regulator, or those little gauze caps on Bunson's burners. It fuses a little above 100° F. Glass vessels, when placed in the bath at that temperature, are not liable to crack, and if it should be spilled, it is not liable to grease everything that it touches. The vessel used as a bath is also very readily cleansed with benzole.

Filtering paper drawn through melted paraffin, left in contact with oil of vitriol, does not change. Labels on acid bottles, after being covered with a gloss of mucilage and dried, are painted over with a coat of paraffin heated somewhat above its freezing point, so as not to give too thick a coating.

Hydrofluoric acid may be kept in glass bottles coated on the inside with paraffin, which coating is formed by rinsing out the dry, warm bottle with paraffin, and chilling it with proper care.

In place of waxed paper, we know of no better substitute for the purpose of covering jars and other like uses, and we should not be surprised to see it come largely in use for preserving fruit, eggs, etc., by giving it a coating sufficient to prevent the action of the atmosphere. Kobell quite lately found a novel application for paraffin, in analysis for excluding the atmosphere from oxidizable solutions, such as ferrous salts, while they are manipulated. Instead of in an atmosphere of hydrogen or carbonic acid gas, a ferrous solution, for instance, is placed in an open dish or beaker in contact with metallic zinc and acid, and covered with a stratum of paraffin, enough heat being applied to keep the latter fluid. Since it has no action on the permanganic solution, the whole may be afterward tested in a beaker without hindrance.

Mohr has for years used paraffin as a protection for corks that are not to be exposed to a higher temperature than the common, but to caustic alkalies and acids. The corks, well selected, are soaked in the melted paraffin as they would be in water. For glass-stoppered alkali bottles, it is likewise an excellent preventive for the fastening of the stoppers and irremediable losses.

Many other applications will suggest themselves to the practical chemist, and we shall be glad to hear more of its application in such and other directions.—*Am. Drug. Cir.*

